# Effect of Different Surface Treatments and Orthodontic Bracket Type on Shear Bond Strength of High-Translucent Zirconia: An In Vitro Study

**DOI:** 10.1155/2022/9884006

**Published:** 2022-06-18

**Authors:** Yasamin Babaee Hemmati, Hamid Neshandar Asli, Mehran Falahchai, Sina Safary

**Affiliations:** ^1^Dental Sciences Research Center, Department of Orthodontics, School of Dentistry, Guilan University of Medical Sciences, Rasht, Iran; ^2^Dental Sciences Research Center, Department of Prosthodontics, School of Dentistry, Guilan University of Medical Sciences, Rasht, Iran; ^3^Dental Sciences Research Center, School of Dentistry, Guilan University of Medical Sciences, Rasht, Iran

## Abstract

**Objective:**

Considering the increasing number of adults seeking orthodontic treatment, and the possible need for bracket bonding to monolithic zirconia restorations, knowledge about the preferred type of bracket (metal/ceramic) and the most efficient surface treatment is imperative to achieve acceptable shear bond strength (SBS). This study aimed to assess the effect of different surface treatments and orthodontic bracket types on SBS of high-translucent zirconia.

**Materials and Methods:**

Totally, 248 disc-shaped zirconia specimens were assigned to two groups for bonding to metal and ceramic brackets. Each group was divided into four subgroups (*n* = 31) for the following surface treatments: no surface treatment (control group), airborne-particle abrasion (APA), tribochemical silica coating with CoJet, and CO_2_ laser irradiation. The mean surface roughness (Ra value) was measured. The SBS was measured after bracket bonding and thermocycling. Data were analyzed by two-way ANOVA, Tukey, Kruskal–Wallis, Mann–Whitney, and Fisher exact tests, and Bonferroni correction (*α*=0.05).

**Results:**

The mean Ra value was significantly different among the surface treatment subgroups (*P* < 0.001). The APA and CoJet subgroups were not significantly different regarding Ra (*P*=0.754). All other pairwise comparisons yielded significant differences (*P* < 0.001). Bracket type, surface treatment, and their interaction significantly affected the SBS (*P* < 0.001). Ceramic brackets bonded to zirconia surfaces treated with CoJet yielded the maximum SBS while ceramic brackets bonded to control and lased surfaces resulted in minimum SBS. No significant difference was noted in the SBS of different surface treatment groups when metal brackets were used (*P* > 0.05).

**Conclusions:**

The use of ceramic brackets and CoJet surface treatment would be the most appropriate combination to achieve optimal bonding to high-translucent zirconia restorations.

## 1. Introduction

The demand for orthodontic treatment is on the rise. In the past, children and young adults were the main candidates for orthodontic treatment; however, an increasing number of adults currently seek orthodontic treatment [1]. In this process, orthodontists increasingly encounter patients with a history of dental procedures such as fixed prosthetic treatments. At present, all ceramic restorations are highly popular due to the increased esthetic demands of patients [2]. Of all ceramic restorations, zirconia restorations are highly popular due to favorable properties such as optimal esthetics, biocompatibility, precise fabrication, high mechanical strength, and optimal dimensional stability [3, 4]. Thus, dental clinicians should have adequate knowledge about how to modify a particular treatment, especially orthodontic treatment, with respect to the existing restorations in the oral cavity to achieve the best possible results.

Bracket bonding is a critical step in orthodontic treatment [3]. The surface to which a bracket is bonded and the bracket type are among the influential factors on bracket bond strength [5, 6]. Zirconia restorations were first used as veneer/core bilayer [7]. However, monolithic zirconia restorations were later introduced to overcome the high frequency of chipping of the veneers, and the problems related to debonding at the zirconia-porcelain interface [8, 9]. New types of monolithic zirconia are currently available to achieve favorable esthetics, translucency, and physical properties, and high-translucent (HT) zirconia is commonly used for monolithic zirconia restorations [10]. Despite the advantages of zirconia, bracket bonding to the zirconia surface is a challenge [3, 4]. The frequency of debonding is higher in bracket bonding to zirconia compared with enamel [5]. Thus, attempts are ongoing to overcome this problem. Moreover, the bond strength should not be too high to damage the enamel or the underlying substrate in the process of bracket removal [3].

Hydrofluoric acid cannot be used for the surface treatment of zirconia since zirconia is devoid of a glass phase [11]. The suggested zirconia surface treatments to enhance the bond strength include airborne-particle abrasion (APA), silica coating, hydrofluoric acid etching, and carbon dioxide (CO_2_), erbium-doped yttrium aluminum garnet, or femtosecond laser irradiation [2, 4]. Of different laser types, the femtosecond laser has shown positive results for this purpose [12]. Nonetheless, it cannot be used in dental clinics and is hardly accessible. Gracia-Sanz et al. [13] in a meta-analysis reported positive results of CO_2_ laser treatment of ceramic surfaces for bonding to composite resin or resin cements. Controversy still exists regarding an ideal zirconia surface treatment to provide adequately high bond strength and minimize the risk of bracket debonding [2]. Studies on bracket bonding to the new types of HT zirconia are limited [14]. Since the phase composition and mechanical properties of these zirconia types are different from those of conventional zirconia [10], their surface treatments may also differ.

In recent years, the demand for invisible orthodontic treatments with the minimal show has increased, which led to the development of ceramic brackets. Considering the differences in properties of ceramic and conventional metal brackets, it is important to find an efficient bonding protocol for them. Information on this topic is limited, and controversy exists regarding the preference for using ceramic brackets on zirconia restoration surfaces [2, 4], which calls for further research in this respect. Considering the significance of surface treatments and bracket type in achieving an optimal bond to zirconia surfaces, the purpose of this study was to assess the effect of different surface treatments and orthodontic bracket types on shear bond strength (SBS) of HT zirconia. The first null hypothesis was that different surface treatments and bracket types would have no significant effect on the SBS of zirconia. The second null hypothesis was that different surface treatments of zirconia would yield similar surface roughness values.

## 2. Materials and Methods

In this study, eight groups were required to measure the SBS of metal and ceramic brackets to zirconia with four surface treatments of control, sandblasting, CoJet, and CO_2_ laser irradiation. To calculate the required sample size, the appropriate effect size had to be considered. Thus, two-way ANOVA feature of GPower 3.1.9.2 software was used for this purpose. Accordingly, the sample size was calculated to be 29 in each group, considering the effect size of 0.25 (medium effect size), study power of 0.90, alpha-0.05, presence of 8 groups, and degree of freedom of 3 (a total of 231). To increase the accuracy of the results, the sample size in each group was increased to 31. Thus, in this in vitro experimental study, 248 disc-shaped specimens with 10 mm diameter and 3 mm thickness were fabricated from monolithic super-translucent and polychromatic yttrium-stabilized tetragonal zirconia (Y-TZP) ceramic (Ceramill Zolid FX Multilayer; Amann Girrbach, Koblach, Austria) by a milling machine (Ceramill, Amanngirrbach, Koblach, Austria). The specimens were sintered at 1450°C according to the manufacturer's instructions and were then polished with 600, 800, and 1200-grit silicon carbide abrasive papers for 15 seconds under water coolant [15]. The ethical committee of Guilan University of Medical Sciences approved the study protocol (IR.GUMS.REC.1399.187).

**Figure 1:**
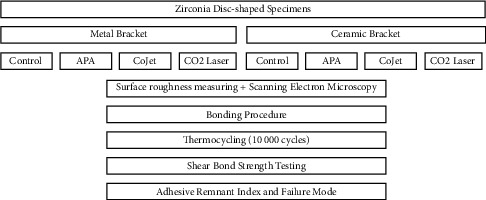
Schematic diagram of experimental procedures (APA: airborne-particle abrasion).

The specimens were cleaned in an ultrasonic bath (96% isopropanol for 3 minutes at room temperature) and dried. They were then assigned to two groups for bonding to metal and ceramic brackets. The specimens in each group were divided into four subgroups (*n* = 31) for the following surface treatments (Figure 1): no surface treatment (control group); APA group in which the specimen surface was airborne-particle abraded with an intraoral sandblaster (Microsandblaster; Dento-Prep Ronvig, Daugård, Denmark) by using 25-µm alumina particles at 10 mm distance for 20 seconds with 0.25 MPa pressure and 90-degree angle [2]; CoJet group in which the specimen surface was airborne-particle abraded by using an intraoral sandblaster (Microsandblaster; Dento‐Prep Ronvig, Denmark) with 30-µm silica-coated alumina particles (CoJet sand, 3 M ESPE, Seefeld, Germany) at 10 mm distance for 20 seconds with 0.25 MPa pressure and 90-degree angle [2, 4]; and laser group in which the specimens were subjected to CO_2_ laser irradiation. For higher energy absorption, the surface was coated with graphite powder (HB pencil) and was then subjected to CO_2_ laser irradiation (DS 10UD, Daeshin, Korea) at 10600 nm wavelength, 4 W power, 159.22 mJ energy density, 50-second irradiation time, and 4 mm focal spot in continuous-wave mode [16, 17]. A noncontact handpiece with no tip was used perpendicular to the surface with a sweeping motion. The surface was cooled with water spray during laser irradiation. All procedures were performed by the same operator.

A profilometer (TR200; Time Group Inc., Beijing, China) was used to assess the surface roughness of specimens after surface treatments. The mean Ra parameter was measured for this purpose. The Ra value was measured at the respective area three times for each specimen with a stylus speed of 0.1 mm/second with 0.001 µm accuracy, and the mean Ra value was calculated for each specimen [18].

A qualitative assessment of the surface was performed by using a scanning electron microscope (SEM). One additional specimen was fabricated for each study group to qualitatively assess the morphological surface changes after each surface treatment under the SEM (Vega, Tescan, Brno, Czech Republic). Micrographs were obtained at ×2000 magnification.

The specimens were cleaned again in an ultrasonic bath (96% isopropanol for 3 minutes at room temperature), rinsed with water, and air-dried. The bonding procedures were performed for the metal and ceramic bracket groups according to the manufacturers' instructions. A thin uniform layer of silane primer containing MDP (Clearfil Ceramic primer, Kuraray Medical, Tokyo, Japan) was applied on the surface by a disposable microbrush as instructed by the manufacturer and dried with gentle air spray. Metal (American Orthodontics, Sheboygan, Washington, USA) and ceramic (Ceramika-I brackets kit MBT .022″ slot, Sklep Falcon Medical Polska, Lodz, Poland) mandibular central incisor brackets were then bonded to the prepared surfaces by a resin cement (GC Ortho Connect, GC Orthodontics, Breckerfeld, Germany). Excess cement was removed by the sharp tip of an explorer, and the resin cement was light-cured at each side of the bracket for 20 seconds with a light intensity of 1400 mW/cm^2^ (Valo Cordless LED Curing Light, Ultradent Products Inc., Utah, USA).

**Figure 2:**
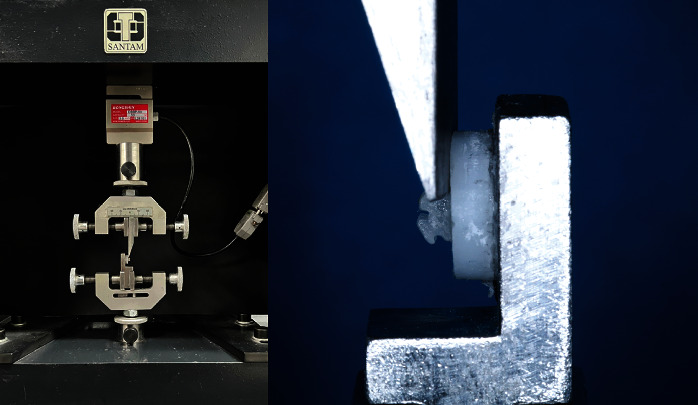
A ceramic bracket bonded to a zirconia disc in a universal testing machine for measurement of shear bond strength.

The zirconia bracket assembly was stored in distilled water at 37°C for 24 hours and then underwent 10,000 thermal cycles between 5 and 55°C with a dwell time of 30 seconds and a transfer time of 20 seconds [14]. The SBS test was then performed by a universal testing machine (Santam, model STM-20, Tehran, Iran; Figure 2). Shear stress was applied downward parallel to the zirconia block surface at a crosshead speed of 0.5 mm/minute until debonding occurred. The SBS was recorded in megapascals (MPa) by dividing the load in Newtons (N) by the bracket base surface area in square millimeters (mm^2^).

The mode of failure was determined under a video measuring machine (VMM; C-Class Vision Measurement Machine; Easson Optoelectronica Technology Co., Suzhou, China). Both bracket and zirconia bonding surfaces were inspected. The adhesive remnant index (ARI) score was calculated as follows to classify the failure mode: [19].

  Score 0: no adhesive remaining on the ceramic surface
  Score 1: less than 50% of adhesive remaining on the ceramic surface
  Score 2: more than 50% of adhesive remaining on the ceramic surface
  Score 3: the entire adhesive remaining on the ceramic surface with the bracket mesh impression on it

Also, the failure mode was categorized into cohesive (fracture within the resin, ceramic structure, or bracket), adhesive (debonding at the resin cement-bracket or resin cement-zirconia interface), and mixed (a combination of adhesive and cohesive failures) types [20].

### 2.1. Statistical Analysis

The normality of data distribution was analyzed by the Shapiro–Wilk test. The homogeneity of variances was analyzed by Levene's test. Since the assumption of homogeneity of variances was not met, the partial eta squared was used to homogenize the variances. The SBS data were analyzed by two-way ANOVA, which was followed by pairwise comparisons with the Tukey test. Since the assumptions were not met for the surface roughness data, the surface roughness of the groups was compared by the Kruskal–Wallis test followed by the Mann–Whitney test with Bonferroni correction for pairwise comparisons. The Fisher exact test was applied to compare the frequency of modes of failure in the groups. All statistical analyses were carried out using SPSS version 25 (SPSS Inc., IL, USA) at a 0.05 level of significance.

## 3. Results

Table 1 presents the surface roughness (Ra) values of zirconia specimens after different surface treatments. The Ra value was significantly different among different surface treatment groups (*P* < 0.001). Pairwise comparisons found no significant difference between APA and CoJet subgroups (*P*=0.754). However, other pairwise comparisons revealed significant differences (*P* < 0.001). The maximum surface roughness value was recorded for specimens subjected to APA and CoJet followed by CO_2_ laser and control groups.

**Figure 3:**
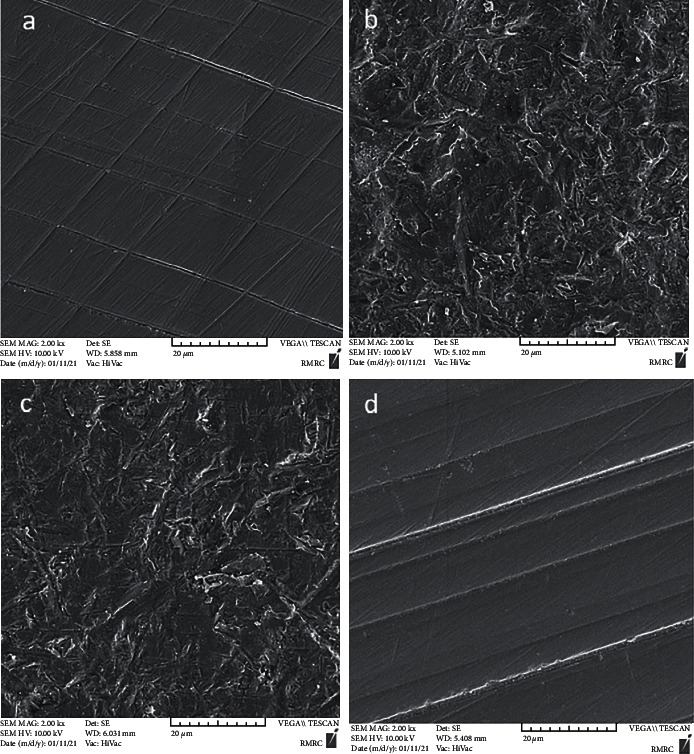
Scanning electron microscopic micrographs from the zirconia surface after different surface treatments at ×2000 magnification. (a) Control; (b) airborne-particle abrasion; (c) CoJet; (d) CO_2_ laser.

SEM assessment of surface changes following different treatments at ×2000 magnification revealed that the polished surface of the control specimen was smoother than other surfaces, and had more superficial vertical and horizontal scratches with slight prominences and depressions. The lased surface had nonhomogeneous and nonuniform scratches. Also, some areas had deep grooves, agglomerates, and a nonhomogeneous surface. Airborne-particle abraded and CoJet-treated surfaces had a wrinkled appearance such that cavitated and porous scratches were noted on the entire surface. These surfaces were much rougher and more heterogeneous than other surfaces. Also, agglomerated and wrinkled areas were seen all over the surface (Figure 3).

Two-way ANOVA was applied to assess the effect of bracket type and different surface treatments on SBS (Table 2). The results indicated significant effects of bracket type, surface treatment type, and their interaction on SBS (*P* < 0.001). Assessment of the effect of bracket type, irrespective of surface treatment type, showed that metal brackets yielded a significantly higher bond strength to zirconia than ceramic brackets. Assessment of the effect of surface treatment, irrespective of bracket type, indicated no significant difference in SBS of APA and CoJet subgroups (*P*=0.169). However, other subgroups had significant differences in SBS (*P* < 0.001). The maximum SBS was noted in CoJet and APA subgroups followed by CO_2_ laser and then the control subgroup.

**Figure 4:**
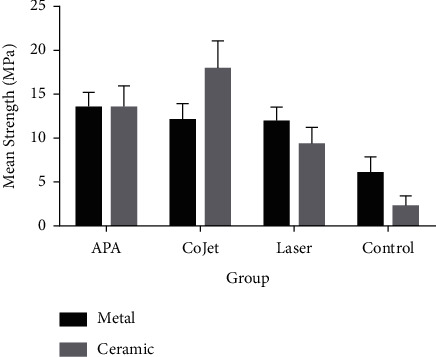
Mean SBS of zirconia (MPa) based on orthodontic bracket type and surface treatment method (APA: airborne-particle abrasion).

The results regarding the interaction effect of bracket type and type of surface treatment on SBS revealed no significant difference between ceramic brackets bonded to airborne-particle abraded surfaces and metal brackets bonded to airborne-particle abraded (*P*=0.999), CoJet-treated (*P*=0.268), or lased (*P*=0.213) surfaces. No significant difference was found between metal brackets bonded to lased surfaces and metal brackets bonded to airborne-particle abraded (*P*=0.270) and CoJet-treated (*P*=0.999) surfaces, or metal brackets bonded to CoJet-treated surfaces and metal brackets bonded to airborne-particle abraded surfaces (*P*=0.332). No other significant differences were noted in pairwise comparisons (*P* > 0.05). The maximum SBS was found in ceramic brackets bonded to CoJet-treated surfaces while the minimum SBS was recorded in ceramic brackets bonded to control surfaces (Figure 4).

Table 3 presents the frequency of ARI scores in the groups. The Fisher exact test found a significant difference in the frequency of ARI scores among the groups (*P* < 0.001). Compared with the groups with ARI score 1 in 100% of the specimens, 29% and 39% of metal bracket/CoJet and ceramic bracket/CoJet specimens showed ARI score 2, respectively, and 23% of ceramic bracket/control specimens showed ARI score 0. Except for the ceramic bracket/control group, in which 23% of specimens showed adhesive failure at the resin cement-zirconia interface, other failures were mixed. No cohesive failure was noted in zirconia specimens or brackets.

## 4. Discussion

Considering the optimal properties of zirconia, it is commonly used as a prosthetic restorative material. [21] Thus, orthodontists may commonly encounter zirconia restorations in patients' mouth. However, zirconia has poorer bonding properties than the enamel, which makes it challenging to obtain adequately high bond strength of orthodontic brackets to zirconia restorations. Studies on tribochemical silica coating for bonding of ceramic brackets are highly limited. [2] Moreover, information regarding the superiority of metal or ceramic brackets, and the surface treatment of choice for bracket bonding to HT zirconia is scarce. The present study revealed the significant effects of orthodontic bracket type and surface treatments on SBS to zirconia. Also, the results indicated that different surface treatments yielded variable surface roughness values. Thus, both the null hypotheses of the study were rejected.

Thermocycling is a type of artificial aging to simulate clinical conditions, which evaluates the resistance of the bond to hydrolysis [14, 22]. In the present study, 10,000 thermal cycles were applied, corresponding to 1 year of clinical service [23]. However, most previous studies on the bond strength of orthodontic brackets to zirconia did not perform aging or applied a lower frequency of cycles. A previous study claimed that by increasing the frequency of the cycles, the clinical setting would be more precisely simulated [24].

Evidence shows that the required bond strength value between orthodontic brackets and the underlying substrate should be 6–8 MPa [25]. The mean SBS to mechanically treated zirconia surfaces in the present study ranged from 11.85 ± 1.66 to 13.33 ± 1.87 MPa for metal brackets, and 9.13 ± 2.07 to 17.87 ± 3.21 MPa for ceramic brackets. Thus, optimal SBS was achieved in all surface treatments. Although the values reported by different studies cannot be precisely compared, the obtained range in the present study was higher than the range reported by some others [26]. One possible reason for this difference may be the use of a primer containing functional monomers in the present study, which was not used in some other studies. The SBS range in the present study was lower than that in two other studies with a methodology close to ours [2, 4] which may be due to the fact that they did not perform thermocycling.

According to the present results, the type of surface treatment significantly affected the SBS of orthodontic brackets to zirconia, which was in agreement with previous findings [2, 4, 14, 19, 24, 25, 27]. In fact, irrespective of bracket type, the specimens that received mechanical surface treatments showed a higher SBS than the control specimens, which may be due to increased surface area as the result of mechanical preparation and enhancement of the bond to MDP [27].

The present study also evaluated the effect of different surface treatments on the zirconia surface quantitatively while very few of the studies on bond strength of orthodontic brackets to zirconia measured the surface roughness of specimens as well. The maximum surface roughness was recorded in the APA and CoJet subgroups followed by CO_2_ laser and control subgroups; these results were also confirmed by SEM assessment of surfaces and agreed with the results regarding the effect of surface treatment on SBS. However, these findings contradicted the results regarding the interaction effect of bracket type and surface treatment on SBS, which may indicate that the SBS does not merely depend on surface roughness created by different mechanical surface treatments, but other factors such as the bracket type also affect it.

CoJet, APA, and CO_2_ laser treatments of zirconia surfaces yielded higher bond strength to ceramic brackets than the control specimens. Higher bond strength in the CoJet compared with the APA subgroup, despite equal surface roughness values, may be due to the use of a primer containing silane and MDP [2] which results in a stronger bond to ceramic brackets in presence of silica particles. The joint effects of 10-MDP and silica-coated surfaces have been previously reported [19, 28]. However, Sanz et al. [2] found no significant difference in SBS between the APA and CoJet groups. Cetik et al. [4] found no significant difference between the APA and Er:YAG laser irradiation. Not using a primer containing functional monomers in the study by Sanz et al. [2], using a different laser type by Cetik et al. [4], and not performing thermocycling by both of them may explain the variations in the results.

Metal brackets had a significantly stronger bond to airborne-particle abraded, CoJet-treated, and lased surfaces than the control specimens in the present study. This finding was in contrast to the results of some studies [2, 20, 27] since they reported that surface treatment with CoJet yielded a higher bond strength than APA. Akay et al. [26] evaluated metal brackets and surface treatments with CoJet and laser. They reported that CoJet yielded a higher bond strength than laser. This difference can be attributed to the use of different laser types, APA and CoJet parameters, study design, methodology, and the materials used.

The result of the comparison of SBS of metal and ceramic brackets in the present study depends on the selected surface treatment, such that ceramic brackets yielded a higher SBS than metal brackets when bonded to CoJet-treated surfaces, compared with other methods. However, the SBS of ceramic brackets to airborne-particle abraded surfaces was comparable to the SBS of metal brackets bonded to surfaces treated with other methods. The SBS of ceramic brackets bonded to lased surfaces was lower than that of metal brackets. The available studies on SBS of metal and ceramic brackets to zirconia surfaces treated with different methods have reported controversial results, which are also different from the present findings. The results reported in such studies did not depend on the type of surface treatment used. Sanz et al. [2] reported higher bond strength of ceramic than metal brackets to zirconia surfaces treated by CoJet, APA, and femtosecond laser. However, Cetik et al. [4] demonstrated that metal brackets bonded to APA and Er:YAG laser-treated zirconia surfaces yielded a higher bond strength than ceramic brackets. Such variations in the results may be attributed to differences in ceramic bracket types, bracket base design, laser types, study design, and not performing thermocycling in such studies. In the present study, metal brackets showed higher SBS to control surfaces than ceramic brackets. This finding may indicate that metal bracket bonding to zirconia is less dependent on mechanical surface treatments and creation of surface roughness. Accordingly, metal brackets showed higher SBS to lased zirconia surface (which had a significantly lower surface roughness than other mechanically treated surfaces) compared with ceramic brackets.

The magnitude and direction of loads applied to zirconia during orthodontic bracket debonding are different from the commonly applied loads to these restorations [27]. Since the goal is to remove brackets without damaging the zirconia restoration, assessment of the mode of failure after debonding is important. Adhesive failure occurs in the case of the presence of a weak bond while cohesive failure occurs in presence of a strong bond [27]. According to the present results, the type of failure was mixed in all test groups, with an ARI score of 1 in most specimens. In the ceramic bracket/CoJet group, 39% of specimens showed an ARI score of 2, which agrees with their higher SBS compared with other groups. ARI score 0 and adhesive failure at the ceramic-resin interface were only noted in the ceramic bracket/control group, which showed the lowest SBS. This result was in agreement with previous findings [2]. Similarly, Ju et al. [14] evaluated the SBS of ceramic brackets bonded to airborne-particle abraded surfaces and reported adhesive failure at the resin-bracket interface in all specimens. The zirconia and bracket surfaces did not show any sign of damage after debonding in the present study, which was in line with the results of Cetik et al. [4] This finding indicates that the SBS did not exceed the optimal threshold.

In brief, the present results may indicate that in presence of zirconia restorations, the bracket material must be carefully selected and a zirconia surface treatment compatible with the bracket type should be chosen to achieve optimally high bond strength. The use of MDP-containing primers should also be carefully considered [29]. Nonetheless, some other factors may also affect the process of orthodontic bracket bonding to zirconia, which should be taken into account, such as the bracket base design, type of adhesive, and type of zirconia [29]. Thus, interpretation of the present findings is limited by the type and brand of zirconia and adhesive, and bracket base design used in this study. Considering the existing limitations, the results of similar future in vitro and clinical studies can further elucidate the mechanism of bonding orthodontic brackets to different types of zirconia. Also, to the best of the authors' knowledge, no previous study is available regarding the effect of bracket base design on bond strength to zirconia. Thus, this topic should be investigated in future studies.

In vitro design was another limitation of this study, since the complex oral conditions cannot be perfectly simulated in vitro. Additionally, some unexplored factors, such as fluid [30] or fluoride [31] contamination may also affect the bond strength, which should be evaluated in future studies. Moreover, despite the positive results of surface treatments applied in the present study with regard to SBS, future studies should focus on the effects of such surface treatments on flexural strength and other properties of different zirconia types. Finally, the conduction of clinical trials can aid in the verification of the present findings.

## 5. Conclusion

Within the limitations of this in vitro study, it may be concluded that the SBS of orthodontic brackets to HT zirconia depends on the type of bracket and surface treatment. Of the groups evaluated in this study, ceramic brackets bonded to CoJet-treated zirconia surfaces yielded the maximum SBS. Nonetheless, no significant difference was noted in the SBS of different surface treatment groups when metal brackets were used.

## Figures and Tables

**Table 1:** Comparison of surface roughness (µm) of specimens subjected to different surface treatments.

Group	Mean ± SD^*∗*^	Statistic^*∗∗*^	*P* value^*∗∗∗*^
Airborne-particle abrasion	0.36 ± 0.06	201	0.001>
CoJet	0.33 ± 0.06		
CO_2_ laser	0.18 ± 0.03		
Control	0.14 ± 0.01		

^*∗*^Standard deviation, ^*∗∗*^ Kruskal−Wallis test, ^*∗∗∗*^*P* < 0.05 was considered statistically significant.

**Table 2:** Effect of orthodontic bracket type and surface treatments on SBS of zirconia using two-way ANOVA.

Variable	Mean of squares		*P* value^*∗*^	Effect size (partial eta square)
Bracket type	1.82	14.98	<0.001	0.059
Surface treatment type	46.74	383.41	<0.001	0.827
Bracket type ^*∗*^ surface treatment type	8.92	73.21	<0.001	0.478

^*∗*^ *P* < 0.05 was considered statistically significant.

**Table 3:** Frequency distribution (percentage) of adhesive remnant index (ARI) scores in the study groups.

Group	MB/C	MB/APA	MB/Co	MB/L	CB/C	CB/APA	CB/Co	CB/L	Total
ARI									
0	0 (0)	0 (0)	0 (0)	0 (0)	7 (23)	0 (0)	0 (0)	0 (0)	7 (3)
1	31 (100)	31 (100)	22 (71)	31 (100)	24 (77)	31 (100)	19 (61)	31 (100)	220 (89)
2	0 (0)	0 (0)	9 (29)	0 (0)	0 (0)	0 (0)	12 (39)	0 (0)	21 (8)
3	0 (0)	0 (0)	0 (0)	0 (0)	0 (0)	0 (0)	0 (0)	0 (0)	0 (0)

MB: metal bracket; CB: ceramic bracket; C: control; APA: airborne-particle abrasion; Co: CoJet; L: laser.

## Data Availability

The datasets used and/or analyzed in the current study are available from the corresponding author on reasonable request.
